# Changing tracks: identifying and tackling bottlenecks in European rail passenger transport

**DOI:** 10.1186/s12544-022-00530-9

**Published:** 2022-03-18

**Authors:** Frank Witlox, Tim Zwanikken, Linde Jehee, Barth Donners, Wijnand Veeneman

**Affiliations:** 1 Department of Geography, Ghent University, Krijgslaan 281, building S8, 9000 Gent, Belgium; 2 Independent consultant, Nijmegen, The Netherlands; 3 Council for the Environment and Infrastructure, The Hague, The Netherlands; 4 Royal HaskoningDHV, Amersfoort, The Netherlands; 5 TU Delft, Delft, The Netherlands

**Keywords:** International passenger rail transport, Bottlenecks to rail transport, Four layer model, Policy recommendations, Europe

## Abstract

For Europe's urban agglomerations to be economically competitive, it is vital that international destinations be easily accessible. Although much has been invested in the construction of European rail infrastructure over the past century, passenger transport by rail has not grown as fast as transport by road and air. So why do people not use international trains more, even though they have an extensive international rail network at their disposal? Based on a series of in-depth interviews with relevant public and private stakeholders and two expert meetings, we identify the main bottlenecks and constraints. In order to understand the complexity of international rail transport, we have divided the existing bottlenecks into four groups corresponding to four layers of the rail transport system: mobility services, transport services, traffic services, and the physical and digital infrastructure. We formulate concrete policy recommendations for improvements to be made in the various components of the rail transport system.

## Introduction

In recent decades, the volume of long-distance travel between Europe's metropolitan agglomerations has increased dramatically, especially by road and air [36, 37]. The spread of the COVID-19 virus has led to unprecedented measures to restrict travel and participation in activities, which has had a huge impact on our hypermobile society [20]. And although the long-term consequences of the observed changes in travel behaviour are not fully known, the most likely changes should result from a shift from onsite to online settings and from regular to more discretionary travel [35]. This will have an impact on the number of (especially long(er) distance) trips, and in the sense that the number of public transport users may be lower (for fear of contamination) than it would have been if the pandemic had never occurred.

International rail services so far account for only a modest share of all trips made [14], which is surprising given that rail transport is a safe and environmentally friendly mode of transport [22, 27]. More so, as the European Commission has already taken many initiatives to improve cross-border rail services—creating a Europe-wide integrated rail network [5, 6, 13]—thus hoping to encourage international travellers to use trains more. European policy in recent decades has focused on the harmonisation of technical systems and safety and operational regulations, and on increasing competition in the market for international passenger rail transport [3, 19, 28]. Despite this, rail's share of the international transport market within Europe has not changed much (remaining at around 7.8%) [12]. The vast majority of travellers still choose to travel by car (or bus) or plane, hence the urgent need for a reassessment of international rail transport policy.

Replacing road and air transport with rail transport still proves difficult in practice because the "intermodal competitiveness" of rail is weak. Choosing to travel by air (despite the "flight shame" or flygskam, see [7]) rather than by train seems an easy choice for travellers because the airline's business model puts the customer first (in terms of price, convenience and comfort). In contrast, the business model of most railways is built around the operation of the railway companies in their domestic transport markets [32].

In this context we formulated two research questions aimed at what is needed to increase the market share of international passenger rail transport. Our first question focuses on what is currently known about international traveler mode choice. This was based on literature study. To improve the position of the international railsector in serving that market, the second question focuses on the obstacles to grow their market share, given the answer to the first question, and what can be done to overcome them. This was based on structured expert consultation. These are the main research questions we aim to answer in this paper.

We concentrate on passenger rail transport, excluding freight. Freight transport, although also relevant, is only discussed in sofar as it affects passenger transport. When studying the bottlenecks, we also start from the perspective of the international rail passenger, and less from the perspective of the railway industry (infrastructure managers, train operators). We limit our geographical scope (and thus also our stakeholder consultation) to the main connections within the international railway networks of Belgium, the Netherlands, France, the United Kingdom, Germany and Austria. We have focused on the main cross-border connections, high-speed rail connections and international night trains. International rail services that are largely of regional interest have not been included.

The paper is structured as follows. In Sect. 2 we briefly discuss the factors influencing modal choice over long distances. In Sect. 3 we discuss the organisational structure of the international rail transport system using a four-layer model as a guide. Section 4 gives a brief overview of stakeholder consultation and data collection. Section 5 discusses the bottlenecks from a passenger perspective and per layer. Section 6 formulates, by way of conclusion, five main policy recommendations. We end with Sect. 7 with a summary of our major findings.

## Factors influencing long distance modal choice

A thorough search of Scopus and Web of Science on international travel mode choice shows very quickly that there has been far less research into the mode choice behaviour of travellers who decide to make long-distance journeys by rail compared to studies that focus on identical journeys by car, plane or coach/bus. The reasons are quite simple: long-distance rail travel—defined here as trips of 100 km or more [4]—is less frequent and, for most people, not part of their daily routines. There is also a problem with (or rather lack of) data availability [15], and the market share of rail in long-distance travel is far below that of air and car [1].

For long-distance travel—based on work by Limtanakool et al. [18] for the car, Creemers et al. [10] for light rail, Vanoutrive et al. [34] for rail, Lannoo et al. [16] and Van Acker et al. [31] for bus/coach travel, Aparicio [1] for car and air travel, and Lepage [17] for rail and air travel—three common mode determining variables are important: (1) travel time, (2) travel cost, and (3) overall comfort/convenience. In most cases, the longer the travel time, the less likely this mode will be chosen. The travel time (door-to-door) consists of the time to reach the means of transport, the waiting time/preparation time, the actual travel time and the time needed to reach the final destination. Each part of the journey is experienced or perceived differently by the traveller and is (generally) considered a disutility (hence the need to reduce travel time). Faster modes win out over slower ones. The combination of the perception of travel time and the actual travel time gives the perceived travel time. This is the time that is taken into account when making a travel decision. Paradoxically, when considering travelling by train, door-to-door time is usually taken into account, but when considering flying, only flight time is often considered (see for example [2, 26]). This makes flying seem more attractive than it really is.

In addition to travel time, travel costs are an important factor in deciding which mode of transport to use. These costs consist of several components (which differ according to the mode), such as fuel, parking and maintenance costs (for car travel), the ticket price and the costs for travelling to and from the station or airport (for rail or air travel). The higher the transport costs, the less likely this mode of transport will be chosen. Cheaper wins from more expensive. As with travel time, the different cost components are perceived differently by the traveller (also depending on his/her price elasticity), but little is known about these cost perceptions for long-distance travel. An important consideration is whether or not the traveller takes all costs (internal and external) into account when deciding on the transport mode to use. Not infrequently the cost of petrol is compared one-to-one with the cost of a public transport ticket, not taking into account parking and maintenance costs of the car.

The comfort level of a means of transport is a third component. It refers to the subjective (even psychological, attitudinal) assessment of the journey. Comfort has to do with convenience, safety and even luxury while "on the road", but also with everything that happens before and after the journey. To illustrate: if it is easier to book online a flight from Amsterdam to South Bolivia—consisting of three to four flights operated by two or three airlines—than a train ticket from Amsterdam to Stockholm, this does not contribute to a high level of comfort. Studies that do take the comfort factor into account show that it is an important factor in the choice of transport mode and that, for example, the higher comfort of an overnight train weighs heavily in its favour compared to car, bus and air travel (e.g. [25]). Higher comfort wins out over lower comfort, but there is also a relationship between comfort level and the perception of travel time: with a higher comfort level, travel time becomes less important. And less comfort at a lower price is often preferred by price-sensitive travellers.

In short, the modal choice behaviour of travellers is not black and white; all the factors discussed above play a role. If a certain mode of transport has a shorter journey time than another, this does not automatically mean that all travellers will base their decision solely on this factor. Empirical data [9, 18] show that travellers for long-distance trips are much more sensitive to the whole range of differences between modes of transport than for local or regional trips.

In order to promote international rail passenger transport, it is important to emphasize competitive journey times and ticket prices, together with the higher level of comfort in comparison with driving or flying as a distinguishing factor. Where international rail transport is not competitive with other modes, measures need to be taken to improve journey times, fares or comfort. To understand how and where these changes can be made, we present a layered model of the rail transport system in the next section.

## The four layers of the rail transport system

**Fig. 1:**
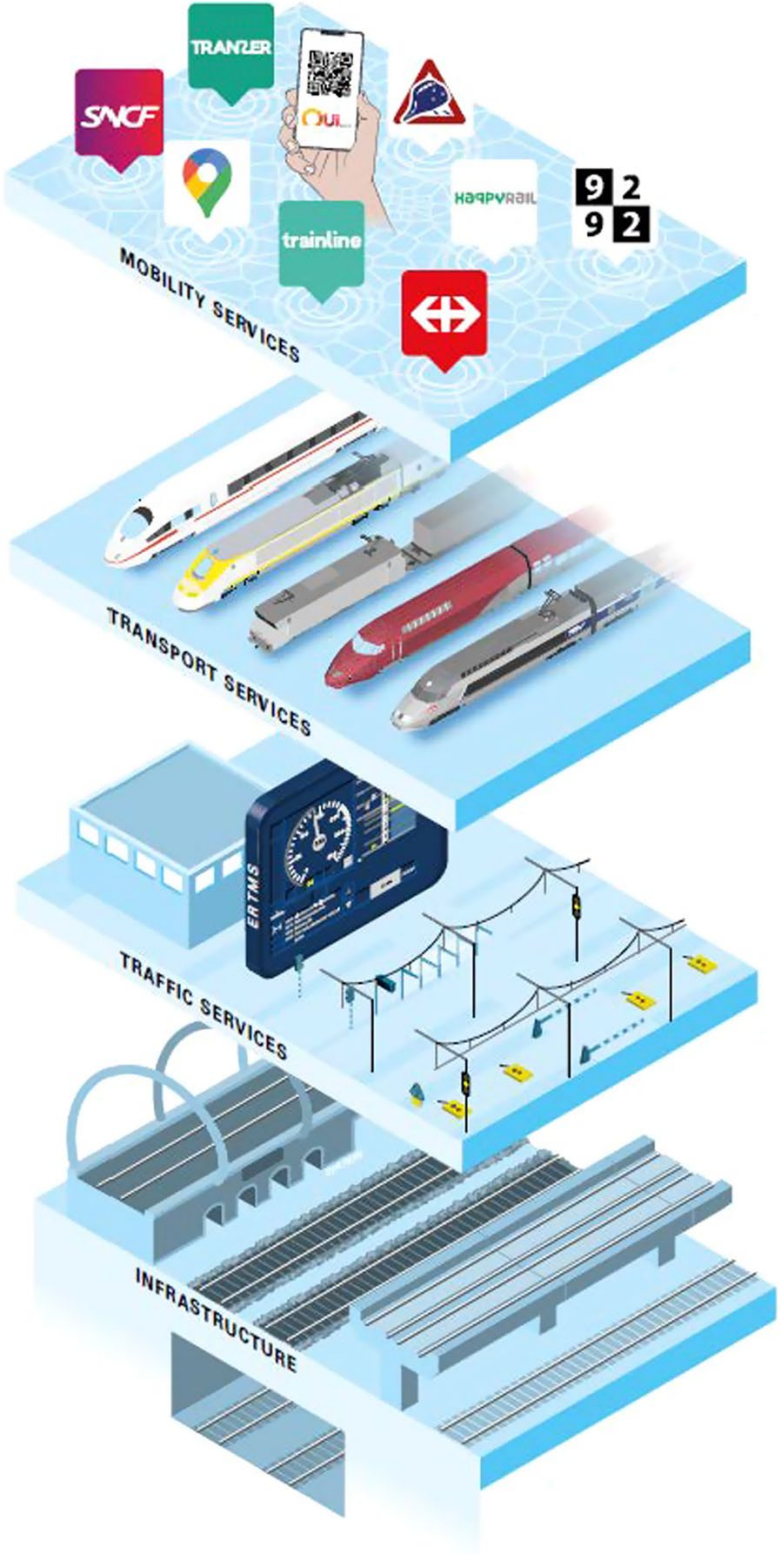
The four layers of the international rail transport system

The world of international rail transport is extremely complex. Not only are there many countries involved, but within each country the operation of international rail services depends on close cooperation between public and private parties. But what exactly is the international rail transport system? To help us understand the rail mobility system, we use the four-layer model [23]. Each layer of this model has its own set of different physical forms and characteristics. It also has its own specific set of players/stakeholders operating within it (see Fig. 1).

Mobility services are the first layer. These consist of a whole range of services with the traveller at the centre to plan, purchase and make their journeys. They include the more or less traditional services such as traffic information and route planners, but also new, often digital services such as real-time route information and Mobility as a Service (MaaS) concepts that are built around the journey from A to B rather than a specific mode of transport. It is about travel. The second layer of the rail transport system is formed by the transport services with the operator at the centre. These concern the operators of the rail transport system (such as railway companies) and the rolling stock and transport services they use to transport their passengers. It is about running trains. The third layer consists of traffic services with the network. This is the set of measures and instruments for optimal and safe use of capacity on the railway network. They include the allocation of train paths to the various operators and traffic management on the available infrastructure, as well as the various systems to promote traffic flows and safety, such as the European Rail Traffic Management System (ERTMS). It is about use of rail capacity. The fourth layer is the physical and digital infrastructure. This is the basis of the rail transport system: the system of tracks, stations, shunting yards and digital hardware on which and along which journeys are made. It is about providing capacity.

The layer model reveals the dependencies between the layers. The lower layers (here layer 4) facilitate what happens in the layers above them. In other words, the layer above makes demands on the delivery of the specific functions and services in the layer below. Without tracks no transport services; without digital infrastructure no train protection system. So without traffic services no transport services, and without transport services no mobility services.

It can be said that each of the four layers of the rail transport system (and its subsystems) is organised in its own way, either between organisations (with vertical separation) or within one organisation (without vertical separation) [21]. A major coordination bottleneck can already be identified here, as each layer has its own organisations and organisational structures. The roles and the division of tasks and responsibilities differ between governments, implementing agencies, transport companies and other commercial partners. The financing, revenue and market models are also very different in each of the layers.

## Stakeholder consultation, expert meeting

To get a full picture of international rail passenger transport, document analyses and a series of consultations and interviews with relevant stakeholders were organized between October 2019 and June 2020. The information collection process is outlined in Table 1. The full list of interviewees can be found in Rli ([24]: pp. 107–110). The interviews and two expert meetings were conducted in face-to-face sessions, as they were organized in pre-COVID times (October 2019 to February 2020). A first round of interviews concentrated on analyzing the international rail system and identifying bottlenecks in the different layers of this system. In total, around 40 people were consulted who have a clear connection to rail transport (as an operator, user/consumer, public authority, business, interest group, academics and/or regional development agency), and who are in competition with rail transport modes (airline/airport operators, bus/coach operators). All in all these interviews were conducted with 7 representatives form transport companies, 8 from infrastructure companies, 13 from local, national and EU government bodies, 2 consultants, 7 scientist and 2 consumer organizations. This was complemented by high-level interviews and document analysis from national ministries, DG Move and European advisory councils in the field of sustainability.

**Table 1:** Information collection process and timing

Planning	Interviews and expert meetings
Summer/autumn 2019	Exploration
	* Explanatory interviews
October 2019 to February 2020	Analysis
	* Interviews
	* Expert meetings
February 2020 to June 2020	Conclusions
	* Additional interviews

**Fig. 2:**
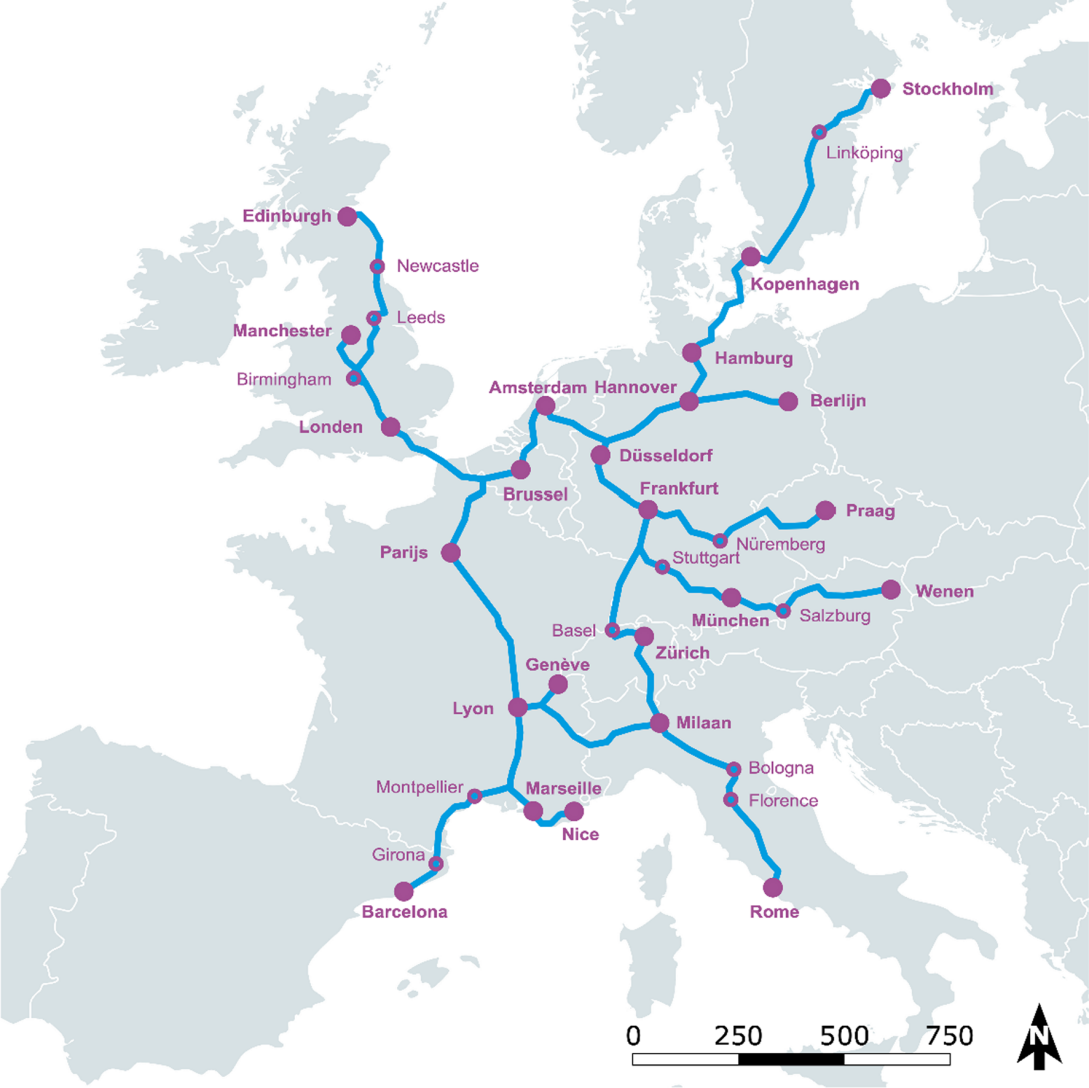
Main international rail transport corridors

The information from these consultations was used as input for two expert meetings, attended by 22 experts (of whom 7 had also been interviewed) (both held on 22 January 2020). The expert meeting served to validate the findings from the consultations. The following stakeholders were present and represent the main international rail transport corridors from Belgium, the Netherlands, France, the United Kingdom, Germany and Austria (see Fig. 2): rail-related (operator/infrastructure) (ALLRAIL; Eurostar; NS International; ÖBB-Personenverkehr AG; ProRail; Thalys; Transdev), consumer-related (European Passengers' Federation; Netherlands Authority for the Consumer and Market; Ombudsman for Public Transport; Rover; Treinreiswinkel; Treinreiziger. nl), air-related (KLM; Royal Schiphol Group), bus-related (FlixMobility), government-related (Federal Council for Sustainable Development Belgium; Province of Limburg; Vereniging Deltametropool), academic/consultant (Arcadis; lynx; TU Delft).

## Bottlenecks

Much of the literature on traveller demand is optimistic about the potential of international rail transport, in terms of stated preference research and transport demand modelling. However, international rail transport in Europe is developing, but arguably not at the pace that the literature on traveller demand suggest. In what follows we report on the main findings (with a strong emphasis on bottlenecks) that emerged from the consultations and expert meetings of representatives of traveler organisations and international travel sectors. We give an overview of the bottlenecks from the passenger's point of view (5.1), and of the bottlenecks related to the four-layer model (5.2).

### Bottlenecks from the passenger’s perspective

The main goal of the European policy to improve international rail services is to optimise the use of the existing railway infrastructure. This is reflected in the two pillars of the European policy: open access and technical harmonisation. The aim of the railway packages is to contribute to the improvement of the quality, competitiveness and efficiency of the European railway sector, the idea being that this will serve the interests of the international rail passenger. However, the assumption that this policy would lead to a large increase in international rail passenger transport has not materialised. In fact, the relative share of rail in the European transport market has actually declined in recent years. This is understandable from the perspective of the international rail traveller, as they still face numerous bottlenecks. It is therefore only logical that travellers do not currently regard the train as a realistic alternative to the car or plane. International travellers are independent and make their own decisions about how to travel; governments can influence that choice by responding to travellers' needs—but have not yet done enough in that respect.

The problem is clearly not limited to the lack of infrastructure alone. International travellers have an extensive European rail network at their disposal. For example, passengers from the Netherlands have direct access to the railway network in Belgium, France and the United Kingdom via the high-speed HSL trains, and to the German railway network via the high-speed ICE trains to Frankfurt and the Intercity to Berlin. These connections give the traveller access to the whole of Europe, albeit with one or more changes. Yet the potential of the train for travellers in Europe is not fully exploited due to at least four major bottlenecks:

#### Journey time: too few fast connections by train

Travel time is an important factor when choosing a mode of transport. The train is more attractive if the travel time is competitive with alternative options. The greatest time savings are achieved when trains run on dedicated tracks, allowing for limited stops and high cruising speed. Many European countries already have such high-speed lines, but on large parts of the infrastructure trains cannot yet run at their maximum cruising speed, either through line speed limitations or additional stops. Moreover, the rail networks have been planned and built to serve national interests, making choices that make perfect sense from a national point of view (such as additional stops to harmonise train speeds and prioritising national services on the line), but resulting in longer travel times for international travellers. A good illustration can be found in the slower operating speeds for high speed trains in the intensively used rail networks of Ile de France, London, Flanders, and the Ruhr area. This means that the travel time savings to international travellers that the European high-speed network provide are curtailed, despite the billions that were invested (including from EU funds).

#### Journey cost: the train is considered/perceived to be expensive

The second barrier experienced by the traveller is the price of the train ticket. Many people consider the price of a train ticket to be high compared to the price of air travel. However, it is by no means certain that this is the case (leaving aside the dumping practices in the low-cost airline sector as a result of COVID-19). It is difficult to accurately compare the cost of train and plane tickets because the prices of the tickets on offer vary so much, but a Consumer Association sample of ticket prices (8) shows that the train is often cheaper than flying for destinations Berlin, London, Prague, and Copenhagen from different cities in the Netherlands. Moreover, travellers often do not take into account the additional costs of travelling by plane, such as airport parking fees and the cost of travelling to and from airports [26].

#### Journey comfort: too few direct train services

Having to change trains during a journey is one of the biggest sources of stress or discomfort for travellers [33]. Changing trains makes a journey more uncertain in many ways. Will I make the connection? What happens if I miss my connection? How will I get from one train to another? Will I be able to wait comfortably for my next train, or will I have to stand on a cold platform? Will the platform be easily accessible [30]. Is it safe or do I have to keep a close eye on my luggage? Moreover, a change almost always involves extra waiting time, making the total travel time from door to door longer than with a direct connection. For some travellers, these objections are so severe that they prefer a longer journey without transferring to a shorter journey with transfer.

Another reason why international travellers are reluctant to choose train journeys with one or more changes is that they feel that passenger rights are inadequate when changes are involved. For example, if your train is delayed and you miss a connection, can you take the next train without having booked a seat? And if there is a delay on part of the journey, can you claim compensation for the whole journey? Notably, services like RailTeam journey planner (http://www.railteam.eu) do provide fairly robust passenger rights for many international journeys with regard to missed connections, but switching from one operator to another can be risky: unlike airlines, operators of rail services have rather limited agreements on taking over each other's passengers in case of delays or cancellations and if it is there, the process to the traveller is far less supported than when missing a flight.

#### Planning and booking of more complex trips is problematic

Another barrier that travellers have to overcome when choosing to travel by train is the poor user friendliness of the booking system compared to other modes of transport. Booking an international train journey is a complicated matter. For many international destinations, it is difficult to find and book tickets. And in many cases tickets are only available three months before the journey, whereas the leisure traveller often wants to plan and book his journey much further in advance. The process leading up to a booking also has its flaws. The service needs to be visible in the set of travel options that can be chosen from, and if travellers do not know what international train services are available, they will never choose the train to get to their foreign destination. Access to the system can be summarised by four key words: knowledge, findability, bookability and security. These factors need to be improved in order to persuade more people to choose the train for their international journeys.

The bottlenecks raised by rail passengers are clearly related to travel time, cost and comfort, in addition to the ease of access to the rail system. In a sense, all these problems relate to layer 1. But since we know that layer 1 is influenced by what may or may not go wrong in layers 2, 3 and 4, we should also look at bottlenecks per layer and between layers.

### Bottlenecks per layer

The passenger perspective showed that mobility services (layer 1) leave much to be desired. There seems to be a clear lack of passenger-friendly access to the system, information and tickets. Travel information (compared to other modes) is poor, booking and rebooking procedures are complicated or impossible, and timetables are unreliable.

The main bottleneck seems to be the booking system. Booking international train journeys is difficult because there is no easy and transparent system for finding and booking international train tickets. Obtaining tickets for main destinations and direct services is reasonably easy, but buying tickets for indirect services or more distant destinations is considerably more complicated. The ticket information landscape is fragmented and technically complex, and the cost–benefit ratio of developing a passenger-friendly platform has so far proved unattractive to potential providers. There are hardly any independent travel information and ticketing providers in Europe. People who want to plan and book a journey get caught up in a confusing maze of rules and information. Railway undertakings generally only offer tickets for their own trains; tickets for other companies' trains, even for connecting trains, are only offered in limited cases (e.g. for services of partner companies). Moreover, each railway undertaking has its own sales channels and digital systems, which means that some tickets can only be purchased online, by phone or at a ticket office.

Finding and buying airline tickets is now much easier, as booking one or more flights is made easy by platforms such as skyscanner, cheaptickets, gotogate, and google flights. When choosing a trip, people are influenced by how easy it is to find and book a trip. If finding a ticket proves difficult, they are likely to choose another travel option for which they can easily obtain a ticket. Airlines work together internationally in broad alliances; railway companies take a different approach. For example, NS (the national railway company of the Netherlands) is not allowed to sell the cheap tickets of Ouigo, a subsidiary of SNCF (the national railway company of France). NS International sells tickets to London and Kent, but not to other destinations in the UK. The national carriers generally do not sell tickets for services of new entrants to the railway market and vice versa. As a result, international train journeys often have to be split up into a series of shorter journeys. This is not only inconvenient to book but also affects passengers' rights in the event of delays or missed connections.

There is no uniform European reservation system for train journeys. In Europe, there are two main systems for reserving trains: In one system, the traveller buys a ticket for a certain route on a certain day, whereby different trains can be used (this flexible system is often referred to as the "German system"). This is advantageous for travellers who miss a connection. In the other system, the traveller buys a ticket for a specific train on a specific day (this inflexible system is often called the "French system"). In this system, the journey must be made at a specific time and seat reservation is mandatory. In practice, it is difficult to combine these two reservation systems. When passengers change from a French to a German reservation, they do not know for sure whether there will be a seat available for them on the connecting train, and if so, in which coach they can find it. And when changing from a German reservation to a French reservation, passengers who miss their connection are not allowed to board the next train (because they did not reserve a seat). None of the providers of tickets for journeys within their own operating area are confronted with this structural defect in the system. The passengers are left to sort it out themselves. There is no direct incentive for the providers of tickets to come up with an overarching system. However, a uniform, public-friendly booking system for international trains would encourage more people to travel by train. As such, this situation represents an indirect, but so far unacknowledged, incentive for greater coordination and cooperation. Greater uniformity of data and data systems across Europe would make access to ticket information and ticketing much easier. It could lead to a system similar to that of the airlines, working on the basis of alliances and code-sharing. However, at the moment it is not in the interest of individual ticket providers to take the initiative in creating such a unified system: the necessary investments are too large and the prospects for recovering the benefits and for third-party entry into the system are too uncertain. Therefore, an umbrella system can only be created by all parties working together.

With regard to transport services (layer 2), but also traffic services (layer 3) and infrastructure (layer 4), there seems to be a strong dominance of national interests through the dominance of national operators and network managers focussing largely on the domestic market. Although some of the first railway lines in Europe were built for cross-border transport, over the past century the rail passenger transport sector has provided value mostly in dense areas and concentrated on transport within national borders. As a result, the interests of international passengers have been under-represented in decision-making.

Over the last decades the international rail services like dwindled. And despite recent examples of a shift, like the ÖBB starting the Nightjet, most players influencing the rail transport system are still nationally oriented. In carrying out their responsibilities, they are nationally oriented: they strive to optimise the national rail transport system without paying much attention to the effects at the international level. COVID-19 also illustrated this. Strong local support for the national railway, less for international connections. They largely neglect the international dimension of the rail transport system because they are 'rewarded' primarily for their domestic performance. For these players, the benefits of international transport performance are mostly intangible and largely irrelevant to performance reviews. The national governments of the Member States underestimate the economic and other interests of metropolitan agglomerations in their decisions on international transport services. In addition, there is a worrying lack of decisive international coordination in striking a balance between the needs of national and international rail transport. This affects the quality and thus the competitiveness of international rail passenger transport.

In addition, the capacity constraints in traffic services (layer 3) are due to a) the different technical, train protection and control systems and the lack of a common language, and b) the problematic distribution of capacity on certain sections between national and international services and between passenger and freight transport. The technical differences between rail systems in Europe have been a major bottleneck for a long time. They concern track gauge, platform height, the electrical power supply and the train control and safety systems. These differences force travellers to transfer or limit operational speed. Technically, the differences between the systems can all be overcome. However, it makes things much more complex for the rolling stock. The consequences are higher costs for the whole system, longer lead times for rolling stock and greater complexity (with the risks involved in obtaining certification).

In relation to infrastructure (layer 4), speed limits are a major bottleneck due to the limited capacity and quality of existing infrastructure and stations. Because the development of the railway infrastructure in the European countries has been concentrated for years on the domestic networks, a fully interconnected international high-speed network has not been established at that spatial scale. This has led to insufficient attention being paid to the development of international high-speed corridors. In the past twenty years, the European Commission has produced four 'railway packages' of legislation for more competition and uniform technical solutions to promote the harmonisation of the railway transport system. However, these European policies have not always led to the desired improvements, either in the market pillar (aimed at open access to rail corridors) or in the technical pillar (aimed at interoperability across the European rail network). Solutions that work well for services to Germany do not work, or work differently, for services to Belgium and France. This can largely be explained by the differences in technical and other systems between the countries, but the considerable differences in culture and procedures also play a role. The railways have a long history, infrastructure managers are considered rather inflexible and operators are not particularly willing to cooperate with each other.

From travel demand research it seems that travellers are positive about international train travel. However, barriers mentioned here hamper the introduction of better services on the main links—the corridors—between Europe's major conurbations, to make good on that promise. Passengers will therefore benefit from a policy focus on making improvements to the core network (which, in principle, can be made quickly). At present, however, policy attention is not focused on these corridors but is spread over the entire network. This situation is at odds with the priorities of the European Commission, which recently set itself the target of completing the core (trans-European transport network) network by 2030 and then the comprehensive network by 2050 [12].

## Five key recommendations for policymakers

With short-haul flights gradually losing public acceptance and increasing road congestion, there is an excellent opportunity to fundamentally rethink the whole issue of international rail travel. Whether COVID-19 will be a game changer here remains to be seen. Van Wee and Witlox [35], in their analysis of the long-term effects of COVID-19 on activity participation and travel behaviour related to rail, warn that investments in capacity expansion of existing connections (such as stations and lines) to reduce congestion (roads) and crowding (public transport) may become less attractive, assuming that attractiveness is based on the difference between social benefits and costs. If bottlenecks are not resolved, it will certainly not help. The bottlenecks within and between the layers of the rail transport system are still approached too little from the perspective of the internal logic of the system itself and too little from the perspective of the international (and domestic) train passenger. In order to have the four layers of the rail transport system cooperate more closely, the functioning of the international corridors must first be improved (Recommendation 1). In addition, improvements are also needed in the four layers of the rail transport system: the mobility services (Recommendation 2), the transport services (Recommendation 3), the traffic services (Recommendation 4), and the infrastructure (Recommendation 5).

### Recommendation 1: work towards a European corridor coordination

A corridor coordinator is needed to ensure that all parties work together to create better connections between international transport links and train paths (i.e. slots allocated to operators to operate services on specific sections of track). Corridor coordination will eventually lead to the demand for a corridor authority. This transport authority within a corridor will ensure that all stakeholders know what level of service is required on each specific section of the corridor (quality of rolling stock, frequency of services and desired speeds). In the long run, the need will arise to merge the corridor authorities into a single European rail traffic management and capacity management system for cross-border services.

The market for international trains has proved difficult to develop. If the policy of open access does not lead to the introduction of the desired international transport services, the corridor coordinator should be able to tender new supranational public service obligations for major routes. Ultimately, this could be carried out by the new corridor authority. An even better service could be achieved if the corridor coordinator were later given the task of increasing the operating speed of the services by solving technical problems and removing infrastructure bottlenecks. European funds can be called upon to provide all or part of the necessary financing. The experience of the Rhine Commission [11] could be helpful in defining the exact tasks and competences of the corridor authority.

### Recommendation 2: mobility services—improve information provision, ticketing and passenger rights

The European rail transport policy must ensure that the rail market is opened up to different transport services and that trains run faster. The train passenger should benefit from this. But if the desired train connection cannot be found or booked, much of the benefit is lost. Much remains to be done in this regard. Specifically, travel and passenger information must become more accessible and international journeys much easier to book. International train travellers want a user-friendly booking procedure that brings together the services and prices of all train operators on a route (former public operators and new market entrants) in one convenient format. As travellers do not travel from station to station but from door to door, they want integrated travel advice and ticketing (cf. MaaS) [29]. There are already a number of apps in development that will provide integrated travel information and ticketing services. However, app-builders face the problem of insufficient access to travel information, passenger data and ticketing because operators do not make them available. Operators must make this data public.

All operators should allow third parties (not just former public operators) to sell tickets. The aim should be to standardise the format of ticket information and conditions, along the lines of the airline situation. This will make it easier, more attractive and economically viable for all train companies, online ticketing platforms and travel agencies to sell third-party tickets. None of the individual national companies has sufficient financial or other interest in dismantling the status quo, so a cooperative approach is needed. International train tickets are usually only available from three months before the date of travel. This excludes the train for travellers who want to book their journey much further in advance. Operators should be encouraged to make international train tickets available earlier than the current three months before the date of travel. A period of at least nine months should be feasible. When tendering for and awarding concessions and public service obligations, the authorities should raise this with the operators and hold them accountable to the extent possible.

Like airlines, rail operators must make agreements among themselves to take over each other's passengers in the event of delays or cancellation of flights (through ticketing). The European Commission can make binding agreements on this in the Rail Passenger Rights Regulation, which is currently being recast. Unfortunately, our interviews showed that some Member States (under the influence of their railway companies) are watering down the agreements, limiting a stronger traveller focus.

### Recommendation 3: transport services—new international services and the train as an attractive option

Part of the railway network capacity that could be utilised by international services is currently unfilled, mostly situated outside of Europe’s densely populated areas. The EU's open access policy is intended to stimulate market players to use this space. However, this has led to the emergence of only a few new operators, most of whom also operate in regional markets and are in fact subsidiaries of the national operators (Abellio is a subsidiary of NS, Arriva is a subsidiary of Deutsche Bahn, etc.). Successful new entrants to the rail market operate in Italy, Austria and the Czech Republic: NTV/Italo, Westbahn and Regiojet. These and other new operators, united in ALLRAIL (Alliance of Rail New Entrants), point out that the national operators, as former state-owned companies, enjoy much greater market protection. National governments—in defiance of EU policy—tend to protect their national operators from competition. Stimulating new services on a European level, beyond open access is required.

Many international travellers still do not see the train as an attractive form of transport. The opening of the market has apparently not given train operators enough incentive to meet the needs of international travellers. A question that arises is what can be done to make train travel more attractive to passengers. Of course, the operators have an important role to play in marketing their products. As the greening of transport is an important government objective, it is conceivable that the national government could (partially) finance an information campaign to bring international train travel to the attention of the public. In addition, the government could use flanking policy to bring about a shift in passenger numbers to more sustainable modes of transport. An example is the French government that makes its support of Air France dependent on a ban on domestic short-haul flights. Companies and governments can boost the rail market by banning such short flights within Europe for their staff. They could also consider ending the tax deduction for business trips by short-haul flights.

Travellers base their choice of transport partly on the perceived price. As discussed above, train journeys are by no means always more expensive than flying. Nevertheless, attractive pricing might encourage travellers to take the train. A reduction of the VAT rate on train tickets on a European level, which would also contribute to the objectives of the European Green Deal. This also applies to the oft-repeated suggestion of reducing rail access charges levied by infrastructure managers for international long-distance trains. For long-distance connections in Europe, a degressive tariff could be applied: the longer the distance, the lower the charge per distance unit.

### Recommendation 4: traffic services—more efficient capacity allocation and more use of information technology

The management of railway capacity is based on a decades-old system that has been continuously adapted and expanded in a series of incremental improvements. This system does not make it easy to free up more space for international trains. More intelligent use can be made of existing capacity. Such a scheme seems reasonable to plan and fill capacity, but it hinders the flexibility needed to increase the frequency of international services on all international routes. By way of illustration, Eurostar argues that it should be possible in the short term to gain at least 16 min on the journey from Amsterdam to London, and that the five infrastructure managers on the Amsterdam-London route are all seeking to optimise the use of capacity within their own areas. This time saving can be achieved if there is better coordination between the available international timetables. The travel time would then be reduced from 3 h 58 min to 3 h 42 min, which would make the train much more competitive with the plane. If there are questions about the distribution of capacity between freight and passenger transport and/or between national and international connections, these options are not operational but political considerations. If there is a scarcity of capacity, the decision on how to allocate priorities is a political one. On many international services, time could be saved if the timetables allocated by different infrastructure managers were better coordinated. According to Eurostar, just a small variation in the timetable can cut 16 min off the travel time and will make the difference for travellers in choosing between flying or taking the train. In such cases, the coordination body could better balance national and international interests when allocating capacity.

The capacity of the railway network can be significantly increased through better use of information technology, which can make rail transport safer and more reliable. Speeds can also be increased, which would facilitate cross-border rail traffic. The introduction of a uniform electricity supply could also contribute to a more intensive use of the railway network. The EU has chosen to implement the ERTMS system for train control and safety management throughout Europe. However, the problem is complex and difficult to grasp, and many stakeholders are concerned about the slow progress of implementation and rising costs. Here we note that the priority application and harmonisation of information technology on the main railway lines will give a significant boost to international rail transport.

### Recommendation 5: infrastructure—invest in and optimize of transboundary rail connections

Wherever possible, railway capacity should be increased primarily through the use of intelligent safety systems such as ERTMS, which make better use of capacity. However, on some sections physical extension may be necessary. The construction of new infrastructure should be limited to cases where capacity is insufficient or speed is insufficient to meet the required quality standards. Improving the infrastructure is possible and feasible. The unbundling of regional, national and international rail transport on some routes will facilitate traffic flows and allow higher operating speeds.

## Conclusion

Research in international travel, on routes where trains and planes compete, is mainly concerned with modal share and travellers' preferences for both options. These studies show a promising position for rail in the market. But this promise is not being fulfilled. Some research also looks at the development of services and their quality. The research looks at what the reasons are for the low demand. There are clear obstacles in the way the management of the railway sector is structured at national and international level. These barriers manifest themselves in the rail services offered, not only in the transport itself, but also in many other aspects of the travel experience. There is a discrepancy between the promises made in research and the reality on the tracks.

This study aims to broaden the research perspective to understand not only what is promised, but also what opportunities exist to strengthen international rail travel as an alternative for routes up to 800 km. It is based on a broad perspective as presented by relevant stakeholders in the relevant sectors. Moreover, it has a clear starting point in the Netherlands, a country from which long-distance rail journeys are always international journeys. Countries such as France, Germany, Italy, Spain and the UK are indeed developing their high-speed networks on a national scale, without any subset of the barriers described here. However, in other countries such as Austria, Belgium, Denmark and the Netherlands, and thus in the European Union as a whole, these barriers limit the potential of rail transport, despite the promising research on modal preferences.

The barriers presented here are of course based on consultation with a limited number of key stakeholders in this Dutch context. A better understanding of these barriers will help to promote the future of European railways. Therefore, we would like to present this here as a first research agenda to address additional research topics in the field of international travel beyond the current main topics of air travel and mode choice. We propose to extend the analysis to other countries and to explore, beyond stakeholder consultation (and including the views of passengers and potential passengers), the potential of new ways to drive the future of international rail travel in Europe.

## Data Availability

Part of this paper (interviews/stakeholders meeting) can be found at the Council for the Environment and Infrastructure and is downloadable from their website.
